# Gene model for the ortholog of *Glys* in *Drosophila busckii*

**DOI:** 10.17912/micropub.biology.001095

**Published:** 2025-04-14

**Authors:** Anne E. Backlund, Logan Cohen, Jeremy White, Lain Grillo, Tony Ianniello, Amanda Swedrowski, Melinda A. Yang, Jennifer Jemc, Chinmay P. Rele, Laura K Reed

**Affiliations:** 1 The University of Alabama, Tuscaloosa, AL USA; 2 Worcester State University, Worcester MA, USA; 3 University of Richmond, Richmond, VA USA; 4 Loyola University, Chicago, IL USA

## Abstract

Gene model for the ortholog of glycogen synthase ( *Glys* ) in the *Drosophia busckii* Sep. 2015 (UC Berkeley ASM127793v1/DbusGB1) Genome Assembly (GenBank Accession: GCA_001277935.1 ). This ortholog was characterized as part of a developing dataset to study the evolution of the Insulin/insulin-like growth factor signaling pathway (IIS) across the genus *Drosophila* using the Genomics Education Partnership gene annotation protocol for Course-based Undergraduate Research Experiences.

**Figure 1. Genomic neighborhood and gene model for Glys in Drosophila busckii:**
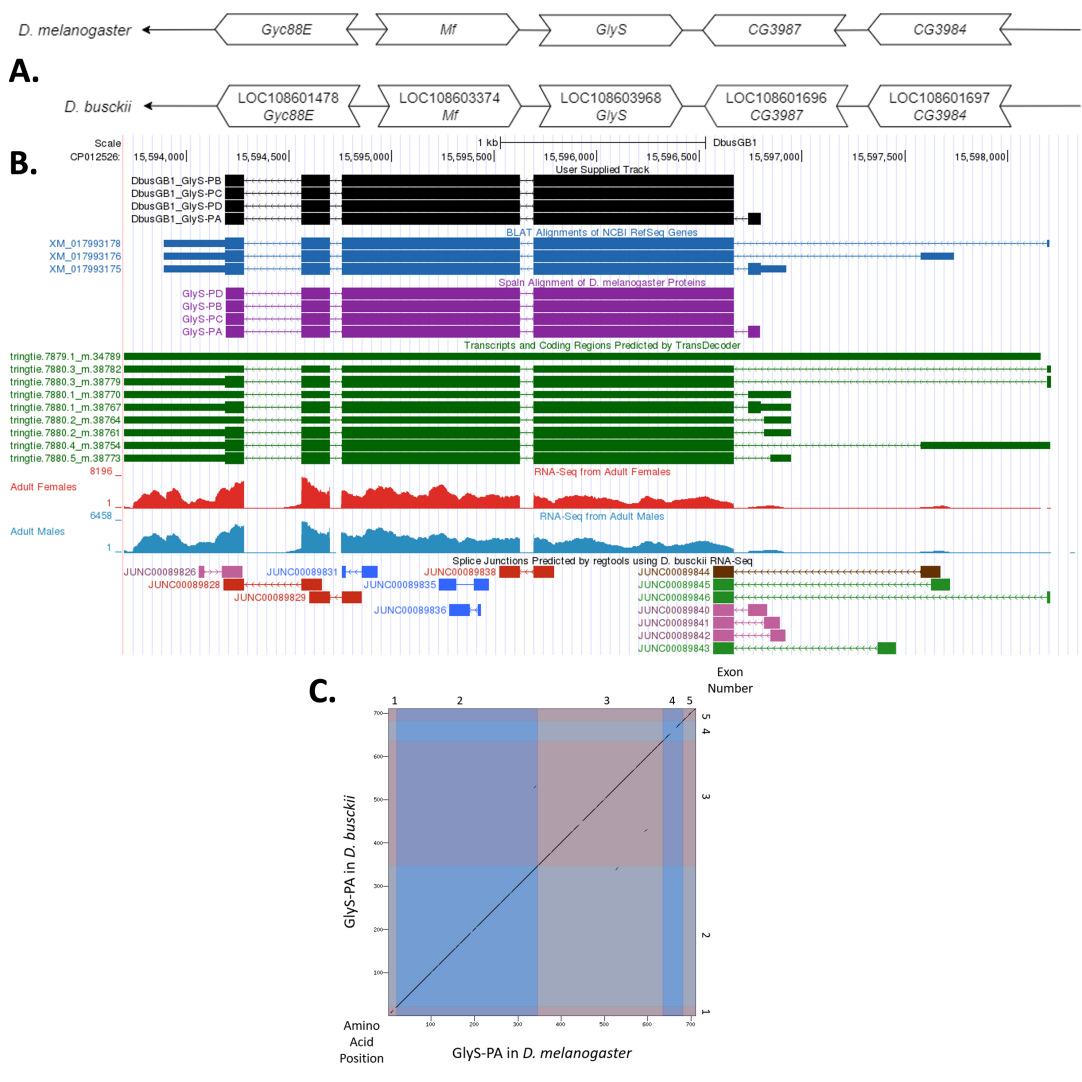
**
(A) Synteny comparison of the genomic neighborhoods for *Glys* in *Drosophila melanogaster* and *D. busckii* .** Thin underlying arrows indicate the DNA strand within which the reference gene– *Glys* –is located in *D. melanogaster* (top) and *D. busckii* (bottom). The thin arrows pointing to the left indicate that *Glys* is on the negative (-) strand in *D. melanogaster* and *D. busckii* . The wide gene arrows pointing in the same direction as *GlyS* are on the same strand relative to the thin underlying arrows, while wide gene arrows pointing in the opposite direction of *Glys* are on the opposite strand relative to the thin underlying arrows. White gene arrows in *D. busckii* indicate orthology to the corresponding gene in *D. melanogaster.* Gene symbols given in the *D. busckii* gene arrows indicate the orthologous gene in *D. melanogaster* , while the locus identifiers are specific to *D. busckii* . **(B) Gene Model in GEP UCSC Track Data Hub** (Raney et al., 2014). The coding-regions of *Glys* in *D. busckii* are displayed in the User Supplied Track (black); CDSs are depicted by thick rectangles and introns by thin lines with arrows indicating the direction of transcription. Subsequent evidence tracks include BLAT Alignments of NCBI RefSeq Genes (dark blue, alignment of Ref-Seq genes for *D. busckii* ), Spaln of *D. melanogaster* Proteins (purple, alignment of Ref-Seq proteins from *D. melanogaster* ), Transcripts and Coding Regions Predicted by TransDecoder (dark green), RNA-Seq from Adult Females and Adult Males (red and light blue, respectively; alignment of Illumina RNA-Seq reads from *D. busckii* ), and Splice Junctions Predicted by regtools using *D. busckii* RNA-Seq ( PRJNA274996 ). Splice junctions shown have read-depths of 10-49, 50-99, 100-499, 500-999, >1000 supporting reads in blue, green, pink, brown, and red, respectively. **(C) Dot Plot of Glys-PA in *D. melanogaster* ( *x* -axis) vs. the orthologous peptide in *D. busckii* ( *y* -axis).** Amino acid number is indicated along the left and bottom; CDS number is indicated along the top and right, and CDSs are also highlighted with alternating colors. Line breaks in the dot plot indicate mismatching amino acids at the specified location between species.

## Description

**Table:** 

* This article reports a predicted gene model generated by undergraduate work using a structured gene model annotation protocol defined by the Genomics Education Partnership (GEP; thegep.org ) for Course-based Undergraduate Research Experience (CURE). The following information in this box may be repeated in other articles submitted by participants using the same GEP CURE protocol for annotating Drosophila species orthologs of Drosophila melanogaster genes in the insulin signaling pathway. * "In this GEP CURE protocol students use web-based tools to manually annotate genes in non-model *Drosophila* species based on orthology to genes in the well-annotated model organism fruitfly *Drosophila melanogaster* . The GEP uses web-based tools to allow undergraduates to participate in course-based research by generating manual annotations of genes in non-model species (Rele et al., 2023). Computational-based gene predictions in any organism are often improved by careful manual annotation and curation, allowing for more accurate analyses of gene and genome evolution (Mudge and Harrow 2016; Tello-Ruiz et al., 2019). These models of orthologous genes across species, such as the one presented here, then provide a reliable basis for further evolutionary genomic analyses when made available to the scientific community.” (Myers et al., 2024). “The particular gene ortholog described here was characterized as part of a developing dataset to study the evolution of the Insulin/insulin-like growth factor signaling pathway (IIS) across the genus *Drosophila* . The Insulin/insulin-like growth factor signaling pathway (IIS) is a highly conserved signaling pathway in animals and is central to mediating organismal responses to nutrients (Hietakangas and Cohen 2009; Grewal 2009).” (Myers et al., 2024). “ *D. busckii* (NCBI:txid30019) belongs to the *Dorsilopha* subgenus (Sturtevant 1942) of the genus *Drosophila* first described by Coquillett in 1901 (Accessed December 22, 2022 from the Integrated Taxonomic Information System (ITIS), https://doi.org/10.5066/F7KH0KBK ). The *Dorsilopha* subgenus is sibling to the *Sophophora* subgenus of *Drosophila* (Remsen and O'Grady 2002). *D. busckii,* possibly native to North America, is cosmopolitan with established populations around the world in primarily temperate regions (CABI Compendium, Accessed December 22, 2022), and has extremely broad host preferences (Sturtevant 1921).” (Backlund et al., 2025).

We propose a gene model for the *D. busckii* ortholog of the *D. melanogaster* glycogen synthase ( *Glys* ) gene. The genomic region of the ortholog corresponds to the uncharacterized protein XP_017848664.1 (Locus ID LOC108603968 ) in the Sep. 2015 (UC Berkeley ASM127793v1/DbusGB1) Genome Assembly of *D. busckii* ( GCA_001277935.1 ). This model is based on RNA-Seq data from *D. busckii* ( PRJNA274996 - Zhou and Bachtrog 2015) and *Glys* in *D. melanogaster* using FlyBase release FB2023_03 ( GCA_000001215.4 ; Larkin et al., 2021; Gramates et al., 2022; Jenkins et al., 2022).

*Glycogen synthase* ( *Glys* ; aka. *GS, GlyS* ) is a gene within the Insulin-signaling pathway in *Drosophila* and encodes a glycosyltransferase that catalyzes linkage of glucose monomers into glycogen. Glys activity is regulated allosterically by glucose 6-phosphate and phosphorylation/dephosphorylation allowing for control of cellular glycogen levels (Plyte et al., 1992; Roach et al., 2012). Null *Glys* mutants exhibit growth defects and reduced larval viability in *Drosophila* (Yamada et al., 2019).

***Synteny***

The reference gene, *Glys,* occurs on chromosome 3R in *D. melanogaster* and is flanked upstream by *Guanylyl cyclase at 88E* ( *Gyc88E* ) and *Myofilin* ( *Mf* ) and downstream by *CG3987* and *CG3984 .* The *tblastn* search of *D. melanogaster* Glys-PA (query) against the *D. busckii* (GenBank Accession: GCA_001277935.1 ) Genome Assembly (database) placed the putative ortholog of *Glys* within scaffold CP012526 at locus LOC108603968 ( XP_017848664.1 )— with an E-value of 0.0 and a percent identity of 96.91%. Furthermore, the putative ortholog is flanked upstream by LOC108601478 ( XP_017844866.1 ) and LOC108603374 ( XP_017847625.1 ), which correspond to *Gyc88E* and *Mf* in *D. melanogaster* (E-value: 0.0 and 0.0; identity: 76.27% and 92.03%, respectively, as determined by *blastp* ; Figure 1A ; Altschul et al., 1990). The putative ortholog of *Glys* is flanked downstream by LOC108601696 ( XP_017845097.1 ) and LOC108601697 ( XP_017845098.2 ), which correspond to *CG3987* and *CG3984* in *D. melanogaster* (E-value: 2e-94 and 5e-58; identity: 45.06% and 35.43%, respectively, as determined by *blastp* ). The putative ortholog assignment for *Glys* in *D. busckii* is supported by the following evidence: The genes surrounding the *Glys* ortholog are orthologous to the genes at the same locus in *D. melanogaster* and local synteny is completely conserved, supported by results generated from *blastp* , so we conclude that LOC108603968 is the correct ortholog of *Glys* in *D. busckii* ( Figure 1A ).

***Protein Model***

*Glys* in *D. busckii* has two unique protein-coding isoforms Glys-PA, and Glys-PB (identical to Glys-PC, and Glys-PD; Figure 1B ). mRNA isoforms *Glys-RB* , *Glys-RC* , and *Glys-RD* contain four CDSs. mRNA isoform *Glys-RA* contains five CDSs. Relative to the ortholog in *D. melanogaster* , the RNA CDS number and protein isoform count are conserved *.* The sequence of Glys-PA in *D. busckii* has 94.64% identity (E-value: 0.0) with the protein-coding isoform Glys-PA in *D. melanogaster* , as determined by *blastp* ( Figure 1C ). Coordinates of this curated gene model are stored by NCBI at GenBank/BankIt (accession **BK065208 , BK065209 , BK065210 , BK065211** ). These data are also archived in the CaltechDATA repository (see “Extended Data” section below).

## Methods

Detailed methods including algorithms, database versions, and citations for the complete annotation process can be found in Rele et al. (2023). Briefly, students use the GEP instance of the UCSC Genome Browser v.435 ( https://gander.wustl.edu ; Kent WJ et al., 2002; Navarro Gonzalez et al., 2021) to examine the genomic neighborhood of their reference IIS gene in the *D. melanogaster* genome assembly (Aug. 2014; BDGP Release 6 + ISO1 MT/dm6). Students then retrieve the protein sequence for the *D. melanogaster* reference gene for a given isoform and run it using *tblastn* against their target *Drosophila* species genome assembly on the NCBI BLAST server ( https://blast.ncbi.nlm.nih.gov/Blast.cgi ; Altschul et al., 1990) to identify potential orthologs. To validate the potential ortholog, students compare the local genomic neighborhood of their potential ortholog with the genomic neighborhood of their reference gene in *D. melanogaster* . This local synteny analysis includes at minimum the two upstream and downstream genes relative to their putative ortholog. They also explore other sets of genomic evidence using multiple alignment tracks in the Genome Browser, including BLAT alignments of RefSeq Genes, Spaln alignment of *D. melanogaster* proteins, multiple gene prediction tracks (e.g., GeMoMa, Geneid, Augustus), and modENCODE RNA-Seq from the target species. Detailed explanation of how these lines of genomic evidenced are leveraged by students in gene model development are described in Rele et al. (2023). Genomic structure information (e.g., CDSs, intron-exon number and boundaries, number of isoforms) for the *D. melanogaster* reference gene is retrieved through the Gene Record Finder ( https://gander.wustl.edu/~wilson/dmelgenerecord/index.html ; Rele et al *.,* 2023). Approximate splice sites within the target gene are determined using *tblastn* using the CDSs from the *D. melanogaste* r reference gene. Coordinates of CDSs are then refined by examining aligned modENCODE RNA-Seq data, and by applying paradigms of molecular biology such as identifying canonical splice site sequences and ensuring the maintenance of an open reading frame across hypothesized splice sites. Students then confirm the biological validity of their target gene model using the Gene Model Checker ( https://gander.wustl.edu/~wilson/dmelgenerecord/index.html ; Rele et al., 2023), which compares the structure and translated sequence from their hypothesized target gene model against the *D. melanogaster* reference gene model. At least two independent models for a gene are generated by students under mentorship of their faculty course instructors. Those models are then reconciled by a third independent researcher mentored by the project leaders to produce the final model. Note: comparison of 5' and 3' UTR sequence information is not included in this GEP CURE protocol.

## Data Availability

Description: GFF, mRNA, and amino acid sequence files. Resource Type: Model. DOI: https://doi.org/10.22002/apf5b-xyg45
